# The predictive value of the triglyceride glucose index combined with cystatin C for the prognosis of patients with acute coronary syndrome

**DOI:** 10.3389/fendo.2024.1423227

**Published:** 2024-08-14

**Authors:** Chen Wang, Pinliang Liao, Chuanqin Tang, Chunlin Chen, Xiaoyu Zhang

**Affiliations:** Department of Cardiovascular Medicine, The First Affiliated Hospital of the Army Medical University, Chongqing, China

**Keywords:** triglyceride glucose index, cystatin C, acute coronary syndrome, percutaneous coronary intervention, major adverse cardiovascular event, predictive biomarkers

## Abstract

**Background:**

Recent studies have shown that the triglyceride glucose index (TyG) and cystatin C (CysC) are closely related to cardiovascular disease, but there is limited research on the prognosis of patients with acute coronary syndrome (ACS) after percutaneous coronary intervention (PCI). The aim of this study was to explore the predictive value of the combination of the TyG index and CysC in predicting major adverse cardiovascular events (MACEs) in ACS patients who underwent PCI.

**Methods:**

This retrospective study included 319 ACS patients who underwent PCI. The clinical endpoint was the occurrence of MACEs, including all-cause mortality, heart failure, non-fatal myocardial infarction, target vessel revascularization, and angina requiring hospitalization. Patients were classified into MACEs (65 cases) and non-MACEs (254 cases) groups. Univariate factor and multivariate analysis were used to identify predictors of MACEs. The receiver operating curve (ROC) of the prediction model of MACEs was determined. Additionally, the net reclassification improvement and integrated discrimination improvement indexes were calculated to further assess the additional predictive value of the risk factors for MACEs. Subgroup and interaction analysis between the TyG index combined with CysC and MACEs were conducted in various subgroups. Patients were stratified according to the optimal cutoff point value of the TyG index and the CysC determined by ROC curve analysis. The Kaplan–Meier analysis method was used to construct a survival curve 1 year after PCI.

**Results:**

During a median follow-up period of 14 months, 65 (20.38%) patients had experienced at least one primary endpoint event. Multivariate logistic regression analysis indicated that the TyG index and CysC were independently associated with an increased risk of MACEs after PCI (OR, 2.513, 95% CI 1.451–4.351, P= 0.001; and OR, 4.741, 95% CI 1.344–16.731, P=0.016, respectively). The addition of the TyG index and CysC to the baseline risk model had the strongest incremental effect for predicting MACEs in terms of the C-statistic from 0.789 (95% CI 0.723–0.855, P<0.001) to 0.799 (95% CI 0.733–0.865, P<0.001). Furthermore, Kaplan–Meier analysis demonstrated that a TyG index greater than 9.325 and a CysC value greater than 1.065 mg/ml were significantly associated with an increased risk of MACEs (log‐rank, all P < 0.01).

**Conclusion:**

The TyG index predicts MACEs after PCI in patients with ASC independent of known cardiovascular risk factors. Adjustment of the CysC by the TyG index further improves the predictive ability for MACEs in patients with ACS undergoing PCI. Thus, both of them are expected to become new prognostic indicators for MACEs in patients with ACS after PCI.

## 1 Introduction

Acute coronary syndrome (ACS), as a common cardiovascular disease (CVD), has a critical condition, high mortality rate, and poor prognosis (1). Percutaneous coronary intervention (PCI) can restore effective blood flow reperfusion, improve myocardial ischemia, alleviate clinical symptoms, reduce ischemic complications, and improve survival (2). However, patients with large-scale infarction or those who have not undergone timely revascularization still have a risk of developing acute myocardial infarction (AMI) complications and major adverse cardiovascular events (MACEs) in the short term, and the prognosis is not optimistic (3). Insulin resistance (IR) is not only the main pathogenesis of diabetes but also an important risk factor for the incidence rate and prognosis of CVDs (4). As a new index to evaluate insulin sensitivity, the triglyceride glucose (TyG) index is not only closely related to IR but also to many CVDs. Previous studies have shown that the TyG index is related to coronary artery calcification, atherosclerosis, and symptomatic coronary artery diseases (CADs) (5–7). Cysteine C(CysC) has also been proven to be closely related to CVDs such as peripheral arterial disease, heart failure (HF), and CAD (8, 9). At present, there are few clinical studies on the prediction of postoperative prognosis in patients with initial ACS by the TyG index combined with CysC. Therefore, this study collected first-time ACS cases to explore the relationship between the TyG index combined with CysC levels and disease severity and postoperative clinical outcomes to provide ideas for improving clinical risk stratification and prognosis in patients after PCI.

## 2 Methods

### 2.1 Study population

ACSs are characterized by a sudden reduction in blood supply to the heart and include ST-segment elevation myocardial infarction (STEMI), non-STEMI (NSTEMI), and unstable angina. The criteria for ACS diagnosis are as follows: 1) chest discomfort at rest is the most common presenting symptom of ACS; 2) electrocardiography can distinguish between STEMI and non-ST-segment elevation ACS (NSTE-ACS); and 3) high-sensitivity troponin measurements are the preferred test to evaluate for NSTEMI (10). The inclusion criteria were as follows: 1) over 18 years old; 2) diagnosed with ACS; and 3) patients undergoing PCI for the first time. The exclusion criteria were as follows: 1) patients with severe liver and kidney dysfunction; 2) patients with severe infectious diseases; and 3) patients with concurrent malignant tumors. We used GPower3.1 to calculate the sample size, effect size d=0.5, a=0.05, 1-β=0.8, according to the above calculation formula, the required sample size is 64 people.

**Figure 1:**
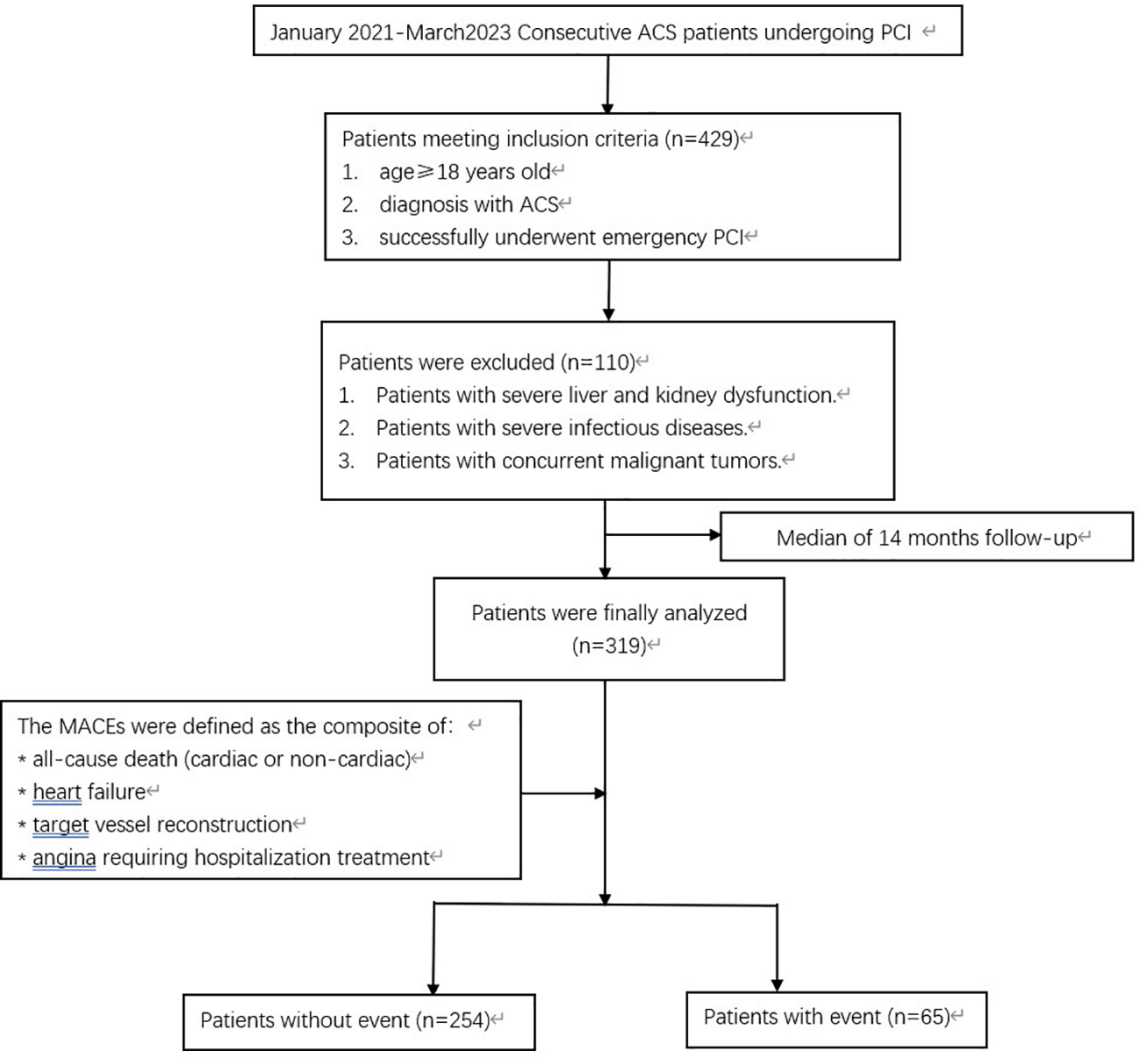
Flow chart of the study population. ACS, acute coronary syndrome; PCI, percutaneous coronary intervention; MACES, major adverse cardiovascular event.

Ultimately, 319 patients who visited the First Affiliated Hospital of the Army Medical University from January 2021 to March 2023 were included in the final analyses. Of these, 252 were males and 67 were females, with ages ranging from 33 to 87 years, with an average age of 61.07 ± 10.94 years. The 319 patients were divided into two groups based on whether MACEs occurred during the observation period: the MACEs group (65 cases) and the non-MACES group (254 cases) (**Figure 1**). This study was approved by the Ethics Committee of the First Affiliated Hospital of the Army Medical University, and all selected research subjects signed informed consent forms.

### 2.2 Data collection and definitions

The patients’ basic clinical data, laboratory indicators, cardiac ultrasound data, and PCI data were collected. The clinical data, such as gender, age, hypertension, diabetes and renal insufficiency history, were collected through a review of the electronic medical record. Hypertension was defined as systolic blood pressure ≥ 140 mmHg and/or diastolic blood pressure ≥ 90 mmHg (11). Diabetes was defined according to one of the following criteria: (1) self-reported diabetes that was previously diagnosed by a physician or the use of glucose-lowering drugs before hospitalization; (2) any one of the characteristic symptoms of diabetes, such as thirst, polyuria, polyphagia, and weight loss with any blood glucose estimation exceeding 11.1 mmol/L; (3) a fasting blood glucose level in excess of 7.0 mmol/L after an overnight fast of 8 h; and (4) a 2-h blood glucose estimation exceeding 11.1 mmol/L after a 75 g glucose load via an oral glucose tolerance test after an overnight fast of 8 h (12). Laboratory indicators of patients before PCI were collected, including urea (Urea), creatinine (Cr), uric acid (UA), cystatin C (CysC), glomerular filtration rate (eGFR), total cholesterol (TC), triglycerides (TGs), high-density lipoprotein cholesterol (HDL-C), low-density lipoprotein cholesterol (LDL-C), glycated hemoglobin, fasting plasma glucose (FPG), and B-type natriuretic peptide precursor (BNP). The TyG index was calculated using the following formula: ln [fasting TG (mg/dL) × FPG (mg/dL)/2]. Measurement of the left ventricular ejection fraction (LVEF) was conducted using echocardiography. Data regarding patient imaging results, time from onset to visit, the location of infarction, and the number of coronary artery lesions during PCI were collected.

### 2.3 Endpoints

The endpoint of this study was major adverse cardiac events(MACEs)at a median 14-month follow-up. The MACEs included the following: all-cause mortality, HF, non-fatal myocardial infarction, target vessel revascularization, angina requiring hospitalization, and HF.

### 2.4 Grouping

Patients were divided into MACEs and non-MACES groups based on whether MACEs occurred during observation; According to cutoff point of the ROC curve, patients were divided into two groups based on the cutoff values for predicting cardiovascular events using the TyG index and CysC: high TyG index group (<9.325), low TyG index group (≥9.325), high CysC group (<1.065), and low CysC group (≥1.065).

### 2.5 Statistical processing

Statistical analysis was conducted using SPSS 23.0 and R Programming Language version 4.0.2 software. Continuous variables were described as mean ± standard deviation and categorical variables were described as counts and percentages. T-tests were used for data that followed a normal distribution, and non-parametric rank sum tests were used for data that did not follow a normal distribution. Comparisons between two groups were conducted using a χ^2^ test. Variable factor and univariate factor logistic regression analyses were used to identify MACEs predictors. Variables with univariate p-values <0.10 were selected for multivariate analysis and are expressed as odds ratios (ORs) with 95% confidence intervals (CIs). The receiver operating characteristic (ROC) curves were used for diagnostic value analysis, and the area under the curve, as measured by the C-statistic, was computed to quantify the predictive power of logistic models for MACEs. Additionally, the net reclassification improvement (NRI) and integrated discrimination improvement (IDI) indexes were calculated to further assess the additional predictive value of the risk factors for MACEs. Survival was graphically represented using Kaplan–Meier curves. Differences in survival rates were compared using the log-rank test. A two-tailed p-value <0.05 was regarded as statistically significant.

## 3 Results

### 3.1 Baseline characteristics of the total population

The median follow-up time for the 319 patients included in the criteria was 14 months. There were 65 cases in the MACEs group, accounting for 20.38% of the total, and 254 cases in the non-MACES group, accounting for 79.62% of the total. The characteristics of the included and excluded patients are compared in **Figure 1**. The baseline characteristics of the total population are summarized in **Table 1**. There were statistically significant differences between the MACEs group and the non-MACES group in diabetes mellitus, renal dysfunction, number of treated vessels, Cr, TG, HDL-C, FPG, CysC, and the TyG index (P<0.05). There were no significant differences between the two groups in terms of age, sex, hypertension, LVEF, UREA, UA, eGFR, TC, LDL-C, HbA1c, BNP, and clinical classification. In brief, compared with participants without MACEs, participants with MACEs had greater proportions of cardiometabolic risk factors, including diabetes mellitus, renal dysfunction, and the number of treated vessels (all P<0.05). In addition, participants with MACEs showed elevated concentrations of Cr, CysC, TG, and FPG, as well as a higher TyG index. Furthermore, participants experiencing MACEs had lower levels of HDL-C (all P<0.05).

**Table 1:** Baseline characteristics of the total population.

Variable	All subjects (n=319)	MACES (n=65)	MACES-free (n=254)	t/X²	P-value
Demographics					
Age, years	61.07 ± 10.94	60.93 ± 10.46	61.63 ± 12.71	-0.463	0.643
Male, n (%)	252(79.00)	55(84.62)	197(77.56)	1.553	0.213
Hypertension, n (%)	209(65.52)	43(66.15)	166(65.35)	0.015	0.904
Diabetes mellitus, n(%)	121(37.93)	37(56.92)	84(33.07)	12.507	0.000
Renal dysfunction, n (%)	22(6.90)	12(18.46)	10(3.94)	17.004	0.000
**Number of treated vessels**				33.081	0.000
1-vessel disease, n (%)	212(66.46)	24(36.92)	188(74.02)		
2-vessel disease, n (%)	95(29.78)	35(53.85)	60(23.62)		
3-vessel disease, n (%)	12(3.76)	6(9.23)	6(2.36)		
Echocardiography					
LVEF(%)	61.41 ± 7.97	60.29 ± 9.79	61.69 ± 7.43	1.076	0.285
Laboratory data					
Urea/(mmol/L)	6.07 ± 3.81	6.24 ± 2.03	6.03 ± 4.15	-0.402	0.688
Cr/(μmol/L)	77.55 ± 22.15	83.51 ± 26.63	76.02 ± 20.64	-2.111	0.038
UA/(μmol/L)	362.53 ± 95.06	366.32 ± 105.98	361.55 ± 92.27	-0.360	0.719
CysC/(mg/L)	0.98 ± 0.32	1.12 ± 0.41	0.94 ± 0.28	-3.349	0.001
eGFR/(ml/min/L)	87.32 ± 21.29	82.98 ± 22.86	88.43 ± 20.82	1.744	0.085
TC/(mmol/L)	4.39 ± 1.25	4.65 ± 1.20	4.32 ± 1.26	-1.868	0.063
TG/(mmol/l)	1.89 ± 1.54	2.74 ± 2.78	1.67 ± 0.90	-3.072	0.003
HDL-C/(mmol/L)	1.08 ± 0.28	1.00 ± 0.25	1.10 ± 0.28	2.535	0.012
LDL-C/(mmol/L)	2.84 ± 1.10	2.89 ± 0.83	2.83 ± 1.17	-0.410	0.682
FPG/(mmol/L)	6.52 ± 2.57	7.53 ± 3.49	6.26 ± 2.21	-2.789	0.007
HbA1c/(%)	6.90 ± 5.00	7.00 ± 1.83	6.65 ± 4.61	-0.609	0.543
BNP/(pg/mL)	138.08 ± 301.90	166.61 ± 321.54	130.78 ± 297.49	-0.852	0.395
TyG index	8.95 ± 0.71	9.32 ± 0.91	8.86 ± 0.62	-4.353	0.000
**Clinical classification**				1.456	0.652
STEMI (n, %)	84(26.33)	14(21.54)	70(27.56)		
NSTE-ACS (n, %)	190(59.56)	38(58.46)	152(59.84)		
UA (n, %)	45(14.11)	8(12.3)	37(14.57)		

Data are shown as mean ± standard deviation (SD) or median (IQR) for continuous variables and proportions (%) for categorical variables. LVEF, left ventricular ejection fraction; Urea, urea nitrogen; Cr, creatinine; UA, uric acid; CysC, cystatinC; eGFR, estimated glomerular filtration rate; TC, total cholesterol; TG, triacylglycerol; HDL-C, high-density lipoprotein cholesterol; LDL-C, low-density lipoprotein cholesterol; FPG, fasting plasma glucose; HbA1c, hemoglobin A1c; BNP, B-natriuretic peptide; TyG index, triglyceride glucose index; STEMI, ST-segment elevation myocardial infarction; NSTEMI, non ST-segment elevation myocardial infarction; UA, unstable angina; MACES, major adverse cardiac events.

### 3.2 Analysis of MACEs risk factors

Univariate and multivariate logistic regression analyses were performed to determine the predictors for MACEs in the overall population (**Table 2**). The risk factors for MACEs were diabetes mellitus, renal dysfunction, Cr, CysC, TG, FPG, and the TyG index, (all P<0.05). Furthermore, we found that a higher TyG index was significantly associated with an increased risk of the primary endpoint in patients in univariate analysis (OR=2.423, 95% CI 1.641-3.577, P=0.000) and in multivariate analysis (OR=2.513, 95% CI 1.451-4.351, P=0.001). Likewise, CysC was significantly associated with an increased risk of MACEs in univariate analysis (OR=5.019, 95% CI 2.184–11.537, P=0.000) and in multivariate analysis (OR=4.741, 95% CI 1.344–16.731, P=0.016). Moreover, the results indicated that the number of treated vessels was significantly associated with MACEs in the patient in univariate analysis (OR=3.608, 95% CI 2.239–5.815, P=0.000) and in multivariate analysis (OR=3.775, 95% CI 2.223–6.411, P=0.000).

**Table 2:** Univariate and multivariate analyses for predictors of MACEs in the overall population.

	Univariate analysis		Multivariate analysis	
	OR (95% CI)	P value	OR (95% CI)	P-value
Demographics				
Age	1.006 (0.981-1.032)	0.642		
Male	0.628 (0.301-1.311)	0.216		
Hypertension	1.036 (0.583-1.842)	0.904		
Diabetes mellitus	2.479 (1.452-4.232)	0.001	1.279 (0.659-2.483)	0.467
Renal dysfunction	5.525 (2.268-13.456)	0	3.515 (1.006-12.278)	0.049
Number of treated vessels	3.608 (2.239–5.815)	0	3.775 (2.223-6.411)	0
Echocardiography				
LVEF(%)	0.979 (0.947-1.012)	0.207		
Laboratory data				
Urea/(mmol/L)	1.013 (0.952-1.077)	0.692		
Cr/(μmol/L)	1.014 (1.003-1.026)	0.017	0.990 (0.972-1.009)	0.293
UA/(μmol/L)	1.001 (0.998-1.003)	0.718		
CysC/(mg/L)	5.019 (2.184-11.537)	0	4.741 (1.344-16.731)	0.016
eGFR/(ml/min/L)	0.988 (0.975-1.001)	0.067		
TC/(mmol/L)	1.218 (0.988-1.502)	0.065		
TG/(mmol/l)	1.511 (1.230-1.857)	0	1.713 (0.984-2.983)	0.057
HDL-C/(mmol/L)	0.230 (0.073-0.727)	0.12		
LDL-C/(mmol/L)	1.051 (0.828-1.334)	0.682		
FPG/(mmol/L)	1.171 (1.666-1.285)	0.001	1.007 (0.878-1.155)	0.92
HbA1c/(%)	1.016 (0.963-1.072)	0.56		
BNP/(pg/mL)	1.000 (1.000-1.001)	0.4		
TyG index	2.423 (1.641-3.577)	0	2.513 (1.451-4.351)	0.001

OR, odds ratio; CI, confidence interval. P values in bold are < 0.05.

LVEF, left ventricular ejection fraction; Urea, urea nitrogen; Cr, creatinine; UA, uric acid; CysC, cystatin C; eGFR, estimated glomerular filtration rate; TC, total cholesterol; TG, triacylglycerol; HDL-C, high-density lipoprotein cholesterol; LDL-C, low-density lipoprotein cholesterol; FPG, fasting plasma glucose; HbA1c, hemoglobin A1c; BNP, B-natriuretic peptide; TyG index, triglyceride glucose index; MACEs, major adverse cardiac events.

### 3.3 Incremental predictive performance of the TyG index and CysC in the risk assessment of MACEs

**Figure 2:**
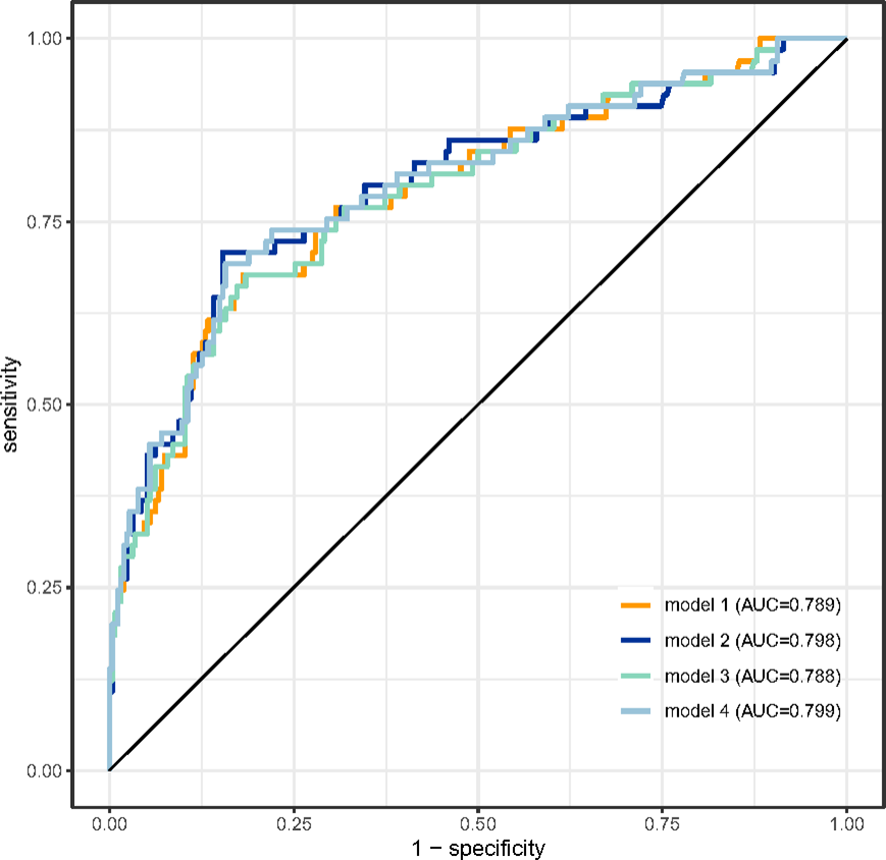
ROC curve analysis of the four models predicting MACEs after PCI. The areas under the ROC curves of Model 1, Model 2, Model 3, and Model 4 for predicting the occurrence of MACEs in ACS patients after PCI were 0.789 (95% CI 0.723–0.855, p<0.001), 0.798 (95% CI 0.732–0.865, p<0.001), 0.788 (95% CI 0.722–0.854, p<0.001), and 0.799 (95% CI 0.733–0.865, p<0.001), respectively. ROC, receiver operating characteristic; MACEs, major adverse cardiac events; PCI, percutaneous coronary intervention; ACS, acute coronary syndrome. CysC, cystatin C; TyG index, triglyceride glucose index.

The synergistic effect of the TyG index and CysC on the prediction of MACEs in patients with ASC undergoing PCI is shown in **Table 3**; **Figure 2**. Compared with the baseline model of established risk factors (Model 1), the addition of CysC (Model 2) significantly increased the C-statistic from 0.789 (95% CI 0.723–0.855, P<0.001) to 0.798 (95% CI 0.732–0.865, P<0.001). The C-statistic of the TyG index (Model 3) was 0.788 (95% CI 0.722–0.854, P<0.001). Moreover, the combination of the TyG index and CysC (Model 4) had the strongest incremental effect for predicting MACEs in terms of the C-statistic from 0.789 (95% CI 0.723–0.855, P<0.001) to 0.799 (95% CI 0.733–0.865, P<0.001), and significantly improved reclassification as assessed by the NRI (0.106, 95% CI −0.163–0.374, P=0.041) and IDI (0.023, 95% CI 0.0004–0.046, P=0.048).

**Table 3:** Evaluation of predictive models for MACEs.

Variables	NRI		IDI		C-statistics	
	Index (95% CI)	*P* value	Index (95% CI)	*P* value	Index (95% CI)	*P* value
Model 1		ref		ref	0.789 (0.723-0.855)	<0.001
Model 2	0.229 (-0.042 - 0.500)	0.098	0.021 (-0.0005 – 0.043)	0.056	0.798 (0.732-0.865)	<0.001
Model 3	0.134 (-0.139 – 0.406)	0.336	0.001 (-0.003 – 0.005)	0.582	0.788 (0.722-0.854)	<0.001
Model 4	0.106 (-0.163 – 0.374)	0.041	0.023 (0.0004 – 0.046)	0.048	0.799 (0.733-0.865)	<0.001

Model 1: Diabetes mellitus + renal dysfunction + vessel disease + Cr + TG.

Model 2: Diabetes mellitus + renal dysfunction + vessel disease + Cr + TG + CysC.

Model 3: Diabetes mellitus + renal dysfunction + vessel disease + Cr + TG + TyG.

Model 4: Diabetes mellitus + renal dysfunction + vessel disease + Cr + TG + CysC + TyG.

MACEs, major adverse cardiac events; PCI, percutaneous coronary intervention; ACS, acute coronary syndrome; Cr, creatinine; TG, triacylglycerol; CysC, cystatin C; TyG index, triglyceride glucose index.

### 3.4 Subgroup analysis

**Figure 3:**
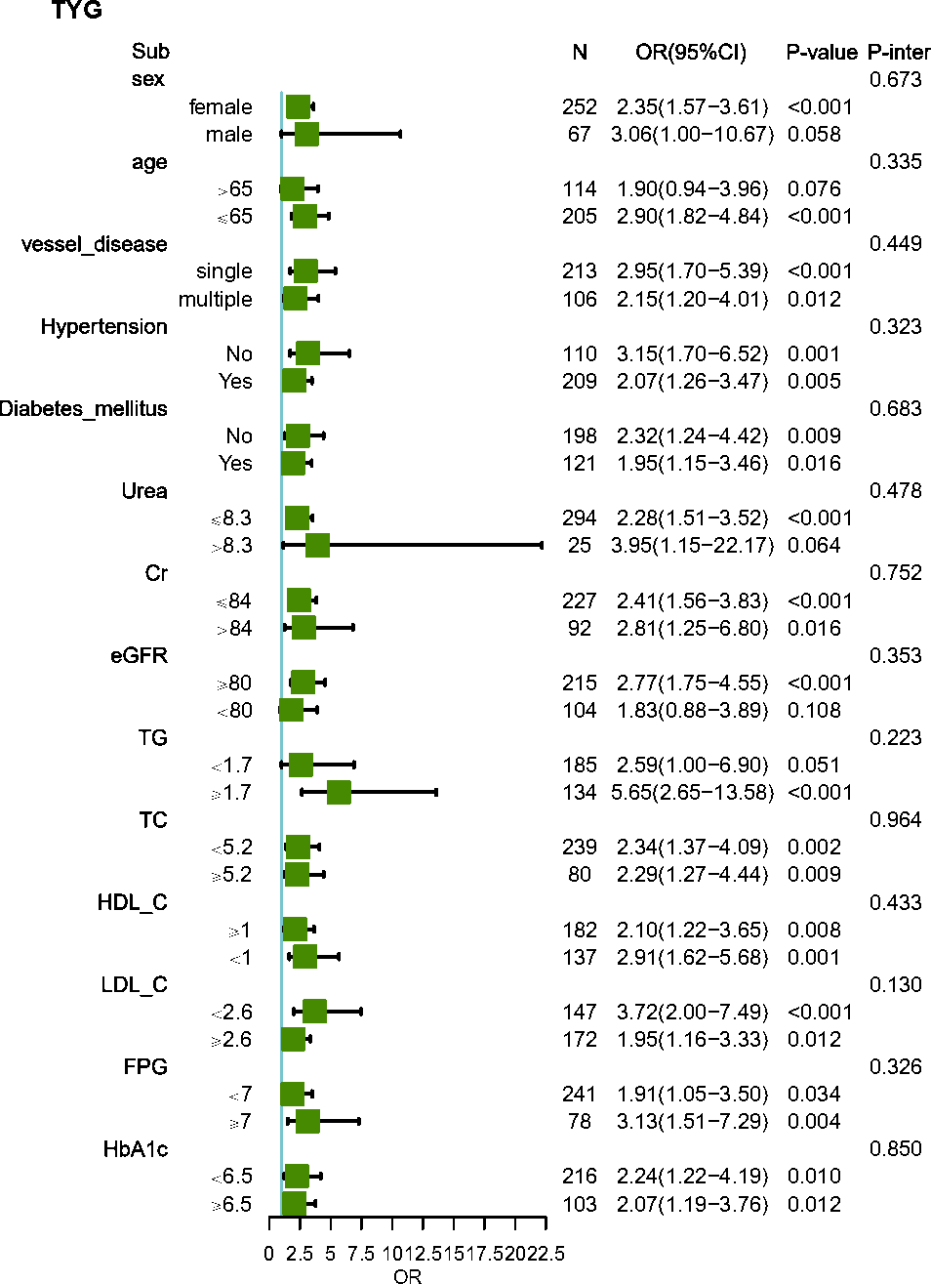
Subgroup and interaction analysis between the TyG index and MACEs across various subgroups. Urea, urea nitrogen; Cr, creatinine; UA, uric acid; eGFR, estimated glomerular filtration rate; TC, total cholesterol; TG, triacylglycerol; HDL-C, high-density lipoprotein cholesterol; LDL-C, low-density lipoprotein cholesterol; FPG, fasting plasma glucose; HbA1c, hemoglobin A1c; TyG index, triglyceride glucose index; CysC, cystatin C.

**Figure 4:**
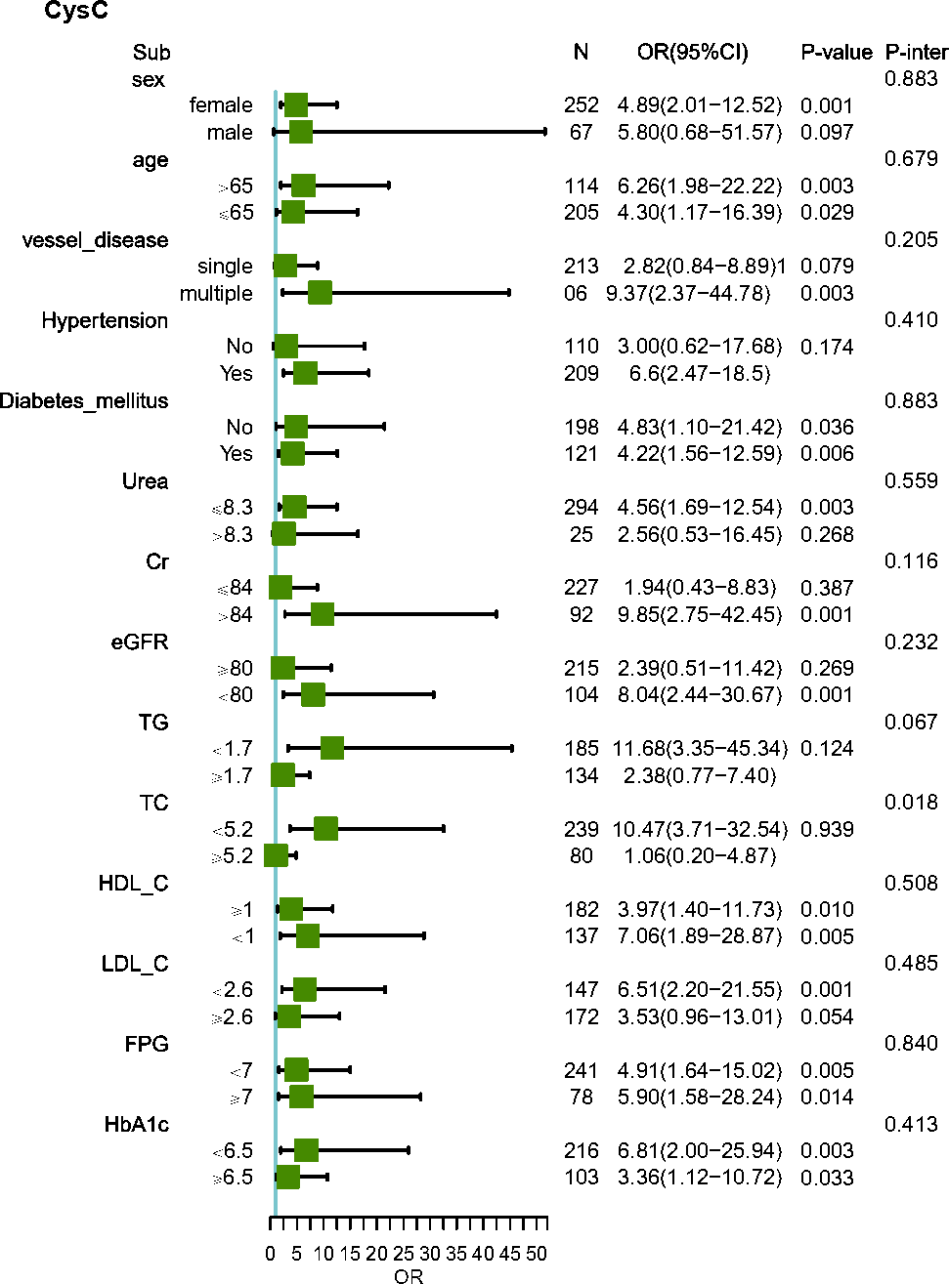
Subgroup and interaction analysis between CysC and MACEs across various subgroups. Urea, urea nitrogen; Cr, creatinine; UA, uric acid; eGFR, estimated glomerular filtration rate; TC, total cholesterol; TG, triacylglycerol; HDL-C, high-density lipoprotein cholesterol; LDL-C, low-density lipoprotein cholesterol; FPG, fasting plasma glucose; HbA1c, hemoglobin A1c; TyG index, triglyceride glucose index; CysC, cystatin C.

The subgroup analysis showed that the association between the TyG index and the risk of MACEs was similar across patient subgroups stratified by age, gender, vessel disease, hypertension, diabetes mellitus, Urea, Cr, eGFR, TG, TC, HDL-C, LDL-C, FPG, and HbA1c (P-values for interaction >0.05) (**Figure 3**). The subgroup analysis showed that the association between Cys C and the risk of MACEs was similar across patient subgroups stratified by age, gender, vessel disease, hypertension, diabetes mellitus, Urea, Cr, eGFR, TG, HDL-C, LDL-C, FPG, and HbA1c (P values for interaction >0.05), The impact of CysC on MACEs is more significant in individuals with low TC levels (**Figure 4**).

### 3.5 Predictive value of the TyG index and CysC for the risk of MACEs

**Figure 5:**
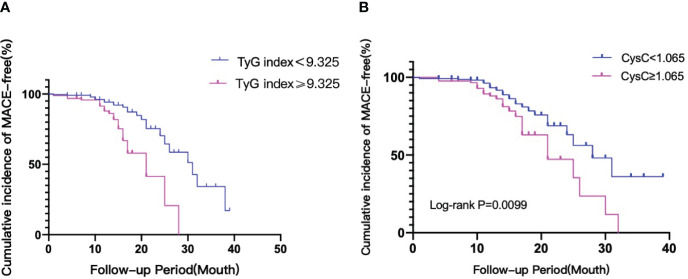
**(A)** The TyG index and risk: Kaplan–Meier curves for the incidences of MACEs. Kaplan-Meier curves of the TyG <9.325 group versus the TyG ≥9.325. **(B)** CysC and risk: Kaplan–Meier curves for the incidences of MACEs. Kaplan-Meier curves of the CysC <1.065 group versus the CysC ≥1.065. TyG index, triglyceride glucose index; CysC, cystatin C; MACEs, major adverse cardiac events.

The Kaplan–Meier analysis revealed that the cumulative incidence of the primary endpoint (MACEs) was significantly higher in patients with a high TyG index (≥9.325, log-rank test, P<0.01) (**Figure 5**). The cumulative incidence of MACEs after PCI was significantly higher in patients with CysC (≥1.065, log-rank test, P<0.01). The risk of developing MACEs significantly increased in the high TyG index and high CysC groups. The risk of MACEs in the high TyG group was 3.279 times higher than that in the low TyG group (HR=3.279, 95% CI 1.938–5.853, P<0.0001). The risk of MACEs in the high CysC group was 1.961 times higher than that in the low CysC group (HR=1.961, 95% CI 1.092–3.522, P=0.0099).

## 4 Discussion

In this study, retrospective analysis demonstrated that, after adjusting for potential confounding factors, the TyG index and CysC remained as independent predictors of MACEs. The TyG index and CysC could independently predict the risk of MACEs in ACS patients after PCI. This study showed that ACS patients with a high TyG index and high CysC levels had a significantly increased risk of MACEs. Additionally, when adding the TyG index and CysC to established risk factors for MACEs, there was a significant improvement in risk prediction in terms of the NRI, IDI, and C statistic. This study demonstrates for the first time that the TyG index combined with the CysC model has a better predictive efficiency for short-term MACEs in ACS patients undergoing PCI. These findings suggested that physicians may apply the TyG index in combination with CysC to identify patients with high residual risk after PCI, and thus they can be subjected to targeted prevention or aggressive treatment to improve their clinical outcomes.

IR is defined as the reduction of metabolic imbalance caused by insulin promoting glucose uptake and utilization efficiency, altering systemic lipid metabolism, and is an indicator of metabolic abnormalities. IR can cause systemic glucose and lipid metabolism imbalance, leading to chronic hyperglycemia and abnormal blood lipids (13, 14). IR may be the most important independent influencing factor leading to coronary atherosclerosis. Further research has found that IR participates in the pathogenesis of CAD: first, high insulin concentration can reduce the production of nitric oxide by activating serum glucocorticoid regulated kinase 1, which in turn will induce matrix protein deposition and fibrosis, and promote coronary atherosclerosis (15). In addition, the activation of glucocorticoid receptors (GR) resulted in the stable DNA demethylation of cytosine in glucocorticoid response elements in the promoter of the liver-specific tyrosine aminotransferase (*TAT*) gene. The elevated glucocorticoids participated in the epigenetic regulation of particular genes associated with programmed CVDs (16). Second, IR can activate signaling pathways such as the protein kinase C pathway and nuclear factor (NF-κB). The pathway promotes the excessive production of reactive oxygen species (ROS), leading to endothelial dysfunction and CVD (17, 18). Zhuang L et al. found that IL-34 deficiency repressed both the canonical and non-canonical NF-κB signaling pathway, leading to a marked reduction in P-IKKβ and P-IκBα kinase levels and the downregulation of NF-κB p65, RelB, and p52 expression, which drove the decline in chemokine CCL2 expression, thus alleviating cardiac remodeling and HF post-ischemia/reperfusion (19, 20).

Furthermore, it was discovered that H_2_S suppressed macrophage inflammation caused by H_2_O_2_ through reducing the activation of the NLRP3 inflammasome, which resulted in the activation of caspase-1, ultimately decreasing the production of mitochondrial ROS (mtROS) (21). Kishi S, et al. showed that IR, a hallmark of type II diabetes mellitus (T2DM) and metabolic syndrome, has been associated with adverse cardiac remodeling and dysfunction (22). Further research has found that IR increases the levels of CaMKIId oxidization and phospholamban/RyR-2 phosphorylation, which results in abnormal intracellular calcium homeostasis and atrial structural remodeling (23).

The hyperinsulinemia glucose clamp test and HOMA-IR are recognized methods for evaluating IR, but due to their high cost, long duration, and complexity, they are difficult to implement in clinical practice (24). Research has found that compared with other IR substitutes such as the Visceral Obesity Index (VAI), the Chinese Visceral Obesity Index (CVAI), Lipid Accumulation Products (LAP), and the triglyceride to high-density lipoprotein cholesterol ratio (TG/HDL-C), the TyG index is most closely associated with the risk of major adverse cardiovascular and cerebrovascular events (MACCEs) in ACS patients undergoing PCI (regardless of whether they have T2DM or not) and provides more valuable information than other IR alternatives (25, 26). The TyG index is a simple, cost-effective, and reliable IR substitute marker and has been proven to be closely related to hyperinsulinemia normal glucose clamp and HOMA-IR results in healthy and diabetic individuals (27).

At present, the TyG index is widely used to assess the risk of CVD and has demonstrated clear correlations among several CVDs, such as ACS, arteriosclerosis, HF, and coronary artery calcification, and newly diagnosed atrial fibrillation (28–31). A meta-analysis showed a higher TyG index was associated with an increased CAD, MI, and CVD incidence compared with a lower TyG index. They observed 35% and 23% increased risks of CAD and CVD, respectively, per 1-unit increase in the TyG index (32). Another meta-analysis also confirmed that compared with patients with the lowest TyG index category, those with the highest category were independently associated with an increased incidence of atherosclerotic CVDs, CAD, and stroke (33). Chen S et al. suggested that IR is an independent predictor of AF, and hospitalized patients with an elevated TyG index had a higher prevalence of AF (34). Moreover, regardless of the presence of T2DM and HF, a higher TyG index equals more adverse outcomes (increased mortality, hospitalization rates, cardiovascular events, and reduced LVEF) (35). Further research by Wang et al. found that a higher risk of incident HF in an American population, After adjusting for potential confounders, 1-SD (0.60) increase in the baseline TyG index was associated with a 15% higher risk of HF development (36). In brief, the TyG index could serve as a simple and cost-effective marker for risk stratification and the early detection of individuals at a higher risk of CVDs.

Numerous studies have shown that TyG is also closely related to ACS. For ACS patients undergoing PCI, the TyG index may be better at predicting postoperative cardiovascular risk than FPG or HbA1C (37). Pang, S showed that the combination of TyG and the GRACE score can improve the predictive value of MACE risk in NSTE-ACS patients 2 years later (38). Yang Y et al. found relatively lower Cu could exacerbate the inflammatory response upon ischemia/reperfusion injury in patients with AMI after PCI, whereas supplementation with Cu could ameliorate tissue damage during this process (39).

However, to the best of our knowledge, no study has reported the predictive value of the TyG index and CysC in patients with ACS undergoing PCI. Therefore, our study compared the effectiveness of the TyG index and CysC to predict the risk of MACEs in patients with ACS undergoing PCI. This study demonstrated for the first time that the TyG index combined with the CysC model has a better predictive efficiency for short-term MACEs in ACS patients after PCI, compared with individual diagnostic models. It is well acknowledged that the early identification of ACS patients undergoing PCI who have a high residual risk for poor prognosis is crucial for making better management decisions to reduce future cardiovascular events.

Patients with chronic kidney disease (CKD) are at a disproportionately high risk of CVD compared with the general population, and are more likely to suffer adverse outcomes from cardiovascular events (40). Serum CysC exists as an inhibitor of cysteine protease in all nucleated cells. CysC protects cells by regulating cell protein hydrolysis, preventing them from being hydrolyzed by inappropriate proteases (41). CysC is synthesized by nucleated cells and released into the bloodstream. It is freely filtered by the glomerulus and almost completely reabsorbed and metabolized by the renal tubules, but it is not secreted. Even if there is a very small change in the eGFR, it may significantly change the serum CysC concentration. Therefore, this alkaline protein becomes a very sensitive renal filtration marker (42). In a large cohort of older adults, Lidgard B et al. showed that lower eGFR was associated with the risk of incident HF and ischemic stroke, and plasma sphingolipids partially mediated the associations between eGFR and incident HF. If so, adjusting plasma sphingolipids may be potential therapeutic options for preventing the development of incident CVD in CKD (43).

CysC is also involved in several CVDs, such as HF, AMI, and coronary heart disease. It mainly damages the cardiovascular system through affecting lipid peroxidation, coagulation function, smooth muscle cell function, and endothelial cell function (44–46). A meta-analysis showed that high levels of CysC in serum may increase the risk of readmission and all-cause mortality in HF patients (47). Navin Suthahar and their team found a strong correlation between CysC and HF in both sexes in a community cohort followed up for 12.5 years, and an increase in CysC may predict early cardiovascular dysfunction in the progression of HF (48). CysC has also been proven to be associated with the onset and prognosis of AMI in patients. Through a 4-year follow-up study in the AMI population, researchers found that CysC may serve as a potential serum predictor of HF after PCI. The probability of MACEs and all-cause cardiovascular mortality in AMI patients with high CysC levels at admission has increased (49). The combined use of CysC and cTnI has a stronger predictive ability for elderly patients with type 2 myocardial infarction than the use of CysC or cTnI alone (50).

At present, the optimal cutoff value for the TyG index has not been unified. In our study, patients with a TyG index exceeding 9.325 and CysC exceeding 1.065 had an increased risk of developing MACEs, indicating this has certain reference value and can serve as an early warning signal to guide the lifestyle of ACS patients who have previously received PCI, as well as to remind clinical doctors to perform early intervention to reduce the incidence of MACEs. We found that the combination of the two is beneficial for risk stratification and prognosis prediction in ACS patients after PCI, which helps the early detection of high-risk patients and adaption of timely treatment strategies.

The TyG index is related to FPG and TG. This study also found that diabetes mellitus, FPG, and TG are the influencing factors of MACEs in ACS patients after PCI. Complications of CVD, such as heart disease, stroke, and hypertension, may also occur after T2DM is diagnosed. Current T2DM management strategies are centered on glycemic control. However, many people either do not achieve proper blood sugar control or experience adverse side effects from blood sugar lowering medications. Therefore, the complexity of T2DM therapy and the high incidence of comorbidities emphasize the importance of prevention. Using a Mendelian randomization (MR) method, they found that the use of lipid management drugs can prevent the occurrence and risk of T2DM. Icosapent ethyl can reduce the risk of CVD by reducing TGs and the risk of T2DM by 53% through the increase in FADS1 expression (51, 52). Lipoprotein lipase can prevent the occurrence of T2DM and CVD by improving IR and insulin sensitivity (53). Therefore, lipid-lowering drugs can be used as the mechanism of CVD and potential intervention targets.

This study has the following limitations: First, no conclusions can be drawn about causality due to the single-center observational design of the study. Second, the exclusion of a considerable number of patients due to missing data and the low incidence of cardiovascular events might have underestimated the effect. Therefore, further investigation and validation via large-sample multicenter studies are needed. Third, the baseline level of the TyG index was derived from TGs and FPG on admission, which could be affected by the use of lipid-lowering and antidiabetic medications during the follow-up period. Fourth, not many indexes were included in the study, which might weaken the results. In the future, more relevant indexes should be included to explore their relationship with diseases. Finally, whether the fluctuation of the TyG index impacts its predictive ability for the prognosis in patients with ACS undergoing PCI requires further investigation.

## 5 Conclusion

In summary, a higher TyG index and CysC were independently associated with an increased risk of MACEs after PCI in patients with ACS. A combination of the TyG index and CysC has incremental prognostic value for the prediction of MACEs. When the TyG index is ≥9.325 or CysC is ≥1.065mg/L, the risk of MACE occurrence increased significantly. These findings suggest that physicians may apply the TyG index in combination with CysC to identify ACS patients with a high residual risk after PCI, and thus they can be subjected to targeted prevention or aggressive treatment to improve their clinical outcomes.
